# 2-Amino-5-methyl­pyridinium nitrate

**DOI:** 10.1107/S1600536812025196

**Published:** 2012-06-13

**Authors:** Xingchen Yan, Yuhua Fan, Caifeng Bi, Jian Zuo, Zhongyu Zhang

**Affiliations:** a Key Laboratory of Marine Chemistry Theory and Technology, Ministry of Education, College of Chemistry and Chemical Engineering, Ocean University of China, Qingdao, Shandong 266100, People’s Republic of China

## Abstract

In the title salt, C_6_H_9_N_2_^+^·NO_3_^−^, the 2-amino-5-methyl­pyridinium cation and the nitrate anion are cyclically linked through pyridinium and amine N—H⋯O hydrogen bonds [graph set *R*_4_^3^(12)]. These units are extended into a zigzag chain structure lying parallel to the *a* axis, through a second cyclic *R*_2_^2^(8) association involving amine N—H⋯O and aromatic C—H⋯O hydrogen bonds to nitrate O-atom acceptors.

## Related literature

For supra­molecular architectures, see: Wang *et al.* (2012 ▶). For the potential of amine derivatives to form metal-organic frameworks, see: Manzur *et al.* (2007 ▶); Ismayilov *et al.* (2007 ▶); Austria *et al.* (2007 ▶). For related structures, see: Nahringbauer & Kvick (1977 ▶); Sherfinski & Marsh (1975 ▶); Zaouali Zgolli *et al.* (2009 ▶); Dai (2008 ▶). For graph-set analysis, see: Etter *et al.* (1990 ▶).
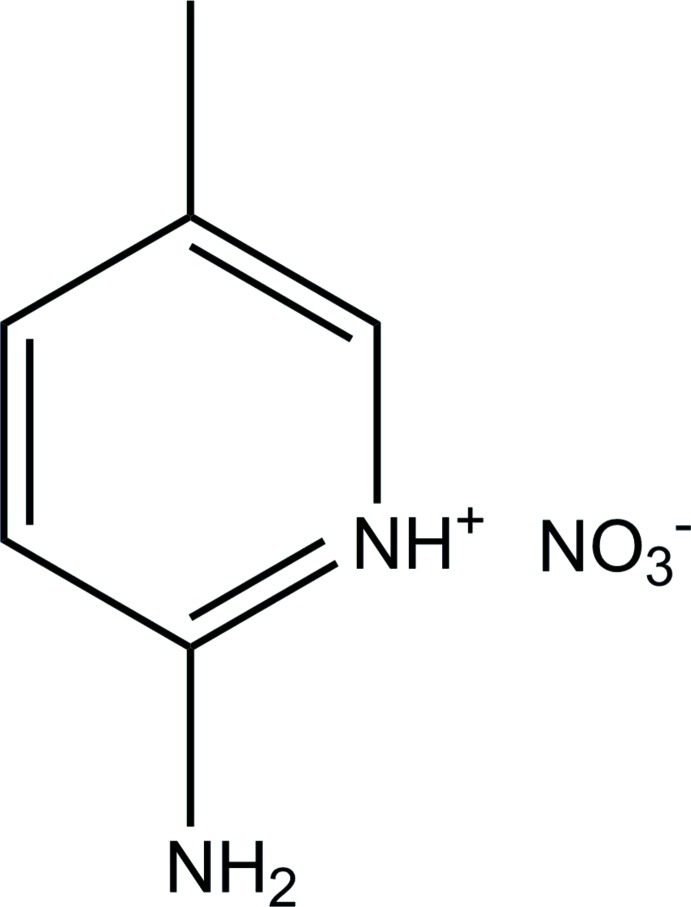


## Experimental

### 

#### Crystal data

C_6_H_9_N_2_^+^·NO_3_^−^
*M* *_r_* = 171.16
Monoclinic, 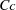

*a* = 8.7711 (7) Å
*b* = 15.7261 (13) Å
*c* = 6.8539 (5) Å
β = 117.455 (2)°
*V* = 838.92 (12) Å^3^
*Z* = 4
Mo *K*α radiation
μ = 0.11 mm^−1^
*T* = 298 K
0.49 × 0.38 × 0.21 mm

#### Data collection

Bruker SMART CCD area-detector diffractometer
Absorption correction: multi-scan (*SADABS*; Sheldrick, 1996 ▶) *T*_min_ = 0.948, *T*_max_ = 0.977
2040 measured reflections
1251 independent reflections
1077 reflections with *I* > 2σ(*I*)
*R*_int_ = 0.031

#### Refinement

*R*[*F* ^2^ > 2σ(*F* ^2^)] = 0.039
*wR*(*F* ^2^) = 0.109
*S* = 1.08
1251 reflections
111 parameters
2 restraints
H-atom parameters constrained
Δρ_max_ = 0.15 e Å^−3^
Δρ_min_ = −0.13 e Å^−3^

### 

Data collection: *SMART* (Bruker, 2000 ▶); cell refinement: *SAINT* (Bruker, 2000 ▶); data reduction: *SAINT*; program(s) used to solve structure: *SHELXS97* (Sheldrick, 2008 ▶); program(s) used to refine structure: *SHELXL97* (Sheldrick, 2008 ▶); molecular graphics: *SHELXTL* (Sheldrick, 2008 ▶); software used to prepare material for publication: *SHELXTL*.

## Supplementary Material

Crystal structure: contains datablock(s) I, global. DOI: 10.1107/S1600536812025196/zs2211sup1.cif

Structure factors: contains datablock(s) I. DOI: 10.1107/S1600536812025196/zs2211Isup2.hkl

Supplementary material file. DOI: 10.1107/S1600536812025196/zs2211Isup3.cml

Additional supplementary materials:  crystallographic information; 3D view; checkCIF report

## Figures and Tables

**Table 1:** Hydrogen-bond geometry (Å, °)

*D*—H⋯*A*	*D*—H	H⋯*A*	*D*⋯*A*	*D*—H⋯*A*
N1—H1⋯O2^i^	0.86	1.95	2.808 (4)	177
N2—H2*A*⋯O1	0.86	2.18	2.992 (4)	157
N2—H2*B*⋯O1^ii^	0.86	2.12	2.948 (4)	160
C2—H2⋯O3^ii^	0.93	2.45	3.304 (4)	153

Symmetry codes: (i) 
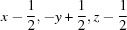
; (ii) 
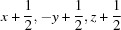
.

**Fig. 1.**
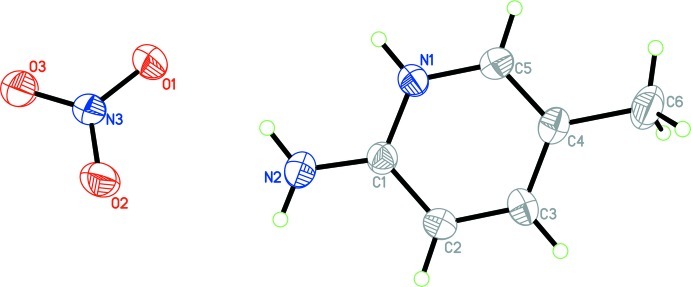
The structure and atom-numbering scheme of the title compound, showing 30% probability displacement ellipsoids.

**Fig. 2.**
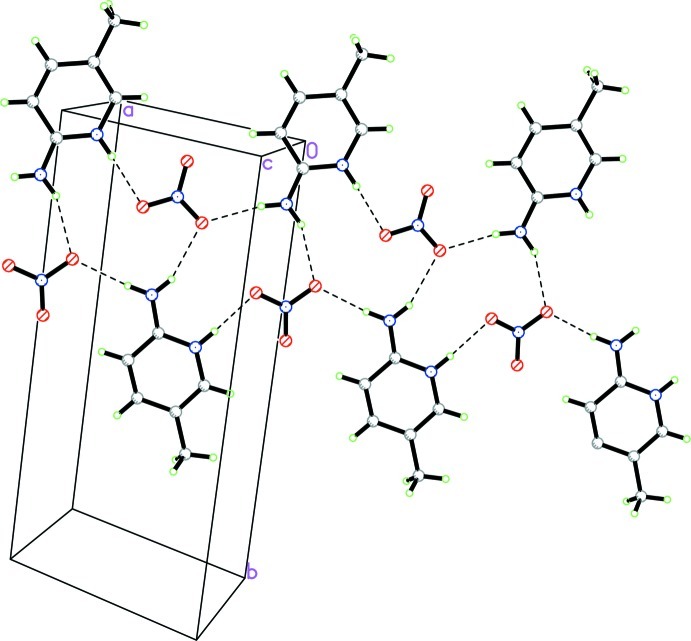
A view of the packing showing the zigzag chain parallel to the a axis. Hydrogen bonds are shown as dashed lines.

## supplementary crystallographic information

### Comment

In the area of predictable assembly of supramolecular architectures, more and more attention has been paid to crystals built from various organic components with specific functional groups (Wang *et al.*, 2012). Because derivatives of the amino acids have the biological activity and amine derivatives have potential to form metal-organic frameworks (Manzur *et al.*, 2007; Ismayilov *et al.*, 2007; Austria *et al.*, 2007), compounds having such functional groups have received considerable attention. The crystal structures of the molecules 2-amino-5-methylpyridine (Nahringbauer & Kvick, 1977), 2-amino-5-methylpyridine hydrochloride (Sherfinski & Marsh, 1975), 2-amino-5-chloropyridinium nitrate (Zaouali Zgolli *et al.*, 2009), and 2-amino-5-cyanopyridinium nitrate (Dai, 2008) have been reported in the literature. We report here the single-crystal structure of the title salt, 2-amino-5-methylpyridium nitrate, C_6_H_9_N_2_^+^ . NO_3_^-^, which was the product obtained in the attempted preparation of a Schiff base Sm^III^ complex using Sm(NO_3_)_3_ . 6H_2_O.

In the title salt (Fig. 1), the 2-amino-5-methylpyridinium cation and the nitrate anion are cyclically linked through pyridinium and amine N—H···O hydrogen bonds [graph set *R*^3^_4_(12) (Etter *et al.*, 1990)] (Table 1). These units are extended into a one-dimensional zigzag chain structure lying parallel to the *a* axis, through a second cyclic *R*^2^_2_(8) association involving amine N—H···O and aromatic C—H···O hydrogen bonds to nitrate O-acceptors (Fig. 2).

### Experimental

2-Amino-5-methylpyridine (0.324 g, 3 mmol) and 1,3-dihydroxyacetone dimer (0.270 g, 1.5 mmol) were dissolved in methanol (10 ml) and this solution was stirred for 6 h at 333 K. Sm(NO_3_)_3_ . 6H_2_O (0.667 g, 1.5 mmol) was then added and the solution was stirred for a further 4 h. This solution was evaporated in air at room temperature, affording pale-yellow needle-shaped crystals suitable for X-ray analysis.

### Refinement

All H-atoms were positioned geometrically and refined using a riding model, with the following constraints: C—H(aromatic) = 0.93 Å, C—H(methyl) = 0.96 Å and N—H = 0.86 Å, with *U*_iso_(H) = 1.2*U*_eq_(N or aromatic C) or *U*_iso_(H) = 1.5*U*_eq_(methyl C).

### Crystal data

**Table:**

C_6_H_9_N_2_^+^·NO_3_^−^	*F*(000) = 360
*M**_r_* = 171.16	*D*_x_ = 1.355 Mg m^−^^3^
Monoclinic, *C**c*	Mo *K*α radiation, λ = 0.71073 Å
Hall symbol: C -2yc	Cell parameters from 1097 reflections
*a* = 8.7711 (7) Å	θ = 2.6–25.8°
*b* = 15.7261 (13) Å	µ = 0.11 mm^−^^1^
*c* = 6.8539 (5) Å	*T* = 298 K
β = 117.455 (2)°	Needle, light-yellow
*V* = 838.92 (12) Å^3^	0.49 × 0.38 × 0.21 mm
*Z* = 4	

### Data collection

**Table:**

Bruker SMART CCD area-detector diffractometer	1251 independent reflections
Radiation source: fine-focus sealed tube	1077 reflections with *I* > 2σ(*I*)
Graphite monochromator	*R*_int_ = 0.031
φ and ω scans	θ_max_ = 25.0°, θ_min_ = 2.6°
Absorption correction: multi-scan (*SADABS*; Sheldrick, 1996)	*h* = −10→10
*T*_min_ = 0.948, *T*_max_ = 0.977	*k* = −15→18
2040 measured reflections	*l* = −8→8

### Refinement

**Table:**

Refinement on *F*^2^	Secondary atom site location: difference Fourier map
Least-squares matrix: full	Hydrogen site location: inferred from neighbouring sites
*R*[*F*^2^ > 2σ(*F*^2^)] = 0.039	H-atom parameters constrained
*wR*(*F*^2^) = 0.109	*w* = 1/[σ^2^(*F*_o_^2^) + (0.0569*P*)^2^ + 0.1897*P*] where *P* = (*F*_o_^2^ + 2*F*_c_^2^)/3
*S* = 1.08	(Δ/σ)_max_ < 0.001
1251 reflections	Δρ_max_ = 0.15 e Å^−^^3^
111 parameters	Δρ_min_ = −0.13 e Å^−^^3^
2 restraints	Extinction correction: *SHELXL*, Fc^*^=kFc[1+0.001xFc^2^λ^3^/sin(2θ)]^-1/4^
Primary atom site location: structure-invariant direct methods	Extinction coefficient: 0.010 (3)

### Special details

**Table:**

Geometry. All e.s.d.'s (except the e.s.d. in the dihedral angle between two l.s. planes) are estimated using the full covariance matrix. The cell e.s.d.'s are taken into account individually in the estimation of e.s.d.'s in distances, angles and torsion angles; correlations between e.s.d.'s in cell parameters are only used when they are defined by crystal symmetry. An approximate (isotropic) treatment of cell e.s.d.'s is used for estimating e.s.d.'s involving l.s. planes.
Refinement. Refinement of *F*^2^ against ALL reflections. The weighted *R*-factor *wR* and goodness of fit *S* are based on *F*^2^, conventional *R*-factors *R* are based on *F*, with *F* set to zero for negative *F*^2^. The threshold expression of *F*^2^ > σ(*F*^2^) is used only for calculating *R*-factors(gt) *etc*. and is not relevant to the choice of reflections for refinement. *R*-factors based on *F*^2^ are statistically about twice as large as those based on *F*, and *R*- factors based on ALL data will be even larger.

### Fractional atomic coordinates and isotropic or equivalent isotropic displacement parameters (Å^2^)

**Table:**

	*x*	*y*	*z*	*U*_iso_*/*U*_eq_	
N1	0.3088 (3)	0.46545 (15)	0.4791 (4)	0.0474 (6)	
H1	0.2362	0.4246	0.4239	0.057*	
N2	0.5229 (4)	0.36493 (16)	0.6464 (4)	0.0593 (7)	
H2A	0.4482	0.3251	0.5882	0.071*	
H2B	0.6289	0.3524	0.7293	0.071*	
N3	0.4592 (3)	0.14462 (15)	0.6312 (5)	0.0583 (7)	
O1	0.3527 (3)	0.19547 (15)	0.5007 (4)	0.0897 (9)	
O2	0.5816 (3)	0.17188 (14)	0.8011 (5)	0.0823 (8)	
O3	0.4431 (3)	0.06826 (15)	0.5931 (5)	0.0912 (9)	
C1	0.4743 (3)	0.44596 (15)	0.6084 (4)	0.0450 (7)	
C2	0.5894 (4)	0.51441 (18)	0.6972 (5)	0.0499 (7)	
H2	0.7054	0.5042	0.7882	0.060*	
C3	0.5302 (4)	0.59519 (18)	0.6493 (5)	0.0534 (8)	
H3	0.6077	0.6399	0.7078	0.064*	
C4	0.3574 (4)	0.61376 (17)	0.5153 (5)	0.0507 (7)	
C5	0.2500 (4)	0.54681 (17)	0.4312 (5)	0.0519 (7)	
H5	0.1340	0.5565	0.3390	0.062*	
C6	0.2926 (5)	0.7045 (2)	0.4632 (7)	0.0767 (11)	
H6A	0.1721	0.7042	0.3623	0.115*	
H6B	0.3539	0.7339	0.3982	0.115*	
H6C	0.3109	0.7328	0.5963	0.115*	

### Atomic displacement parameters (Å^2^)

**Table:**

	*U*^11^	*U*^22^	*U*^33^	*U*^12^	*U*^13^	*U*^23^
N1	0.0453 (13)	0.0430 (12)	0.0475 (15)	−0.0055 (9)	0.0158 (11)	−0.0011 (9)
N2	0.0636 (15)	0.0432 (13)	0.0635 (17)	0.0047 (11)	0.0229 (14)	0.0007 (11)
N3	0.0418 (12)	0.0480 (14)	0.0724 (17)	0.0017 (11)	0.0154 (13)	−0.0003 (13)
O1	0.0638 (16)	0.0579 (14)	0.094 (2)	0.0036 (12)	−0.0086 (14)	0.0108 (13)
O2	0.0610 (14)	0.0595 (14)	0.0835 (17)	0.0097 (11)	−0.0034 (14)	−0.0153 (12)
O3	0.0633 (15)	0.0508 (13)	0.120 (2)	0.0005 (11)	0.0085 (16)	−0.0118 (13)
C1	0.0483 (17)	0.0421 (14)	0.0417 (17)	−0.0008 (12)	0.0183 (13)	−0.0006 (12)
C2	0.0452 (14)	0.0516 (16)	0.0454 (17)	−0.0025 (13)	0.0146 (14)	−0.0027 (13)
C3	0.0578 (17)	0.0450 (15)	0.058 (2)	−0.0104 (12)	0.0268 (16)	−0.0094 (12)
C4	0.063 (2)	0.0416 (15)	0.0499 (18)	0.0049 (13)	0.0278 (16)	0.0012 (13)
C5	0.0469 (16)	0.0508 (15)	0.0534 (19)	0.0053 (13)	0.0190 (14)	0.0059 (12)
C6	0.090 (3)	0.0451 (16)	0.098 (3)	0.0128 (16)	0.046 (2)	0.0119 (17)

### Geometric parameters (Å, º)

**Table:**

N1—C1	1.342 (4)	C2—C3	1.354 (4)
N1—C5	1.362 (4)	C2—H2	0.9300
N1—H1	0.8600	C3—C4	1.394 (4)
N2—C1	1.331 (4)	C3—H3	0.9300
N2—H2A	0.8600	C4—C5	1.351 (4)
N2—H2B	0.8600	C4—C6	1.516 (4)
N3—O3	1.223 (3)	C5—H5	0.9300
N3—O2	1.241 (3)	C6—H6A	0.9600
N3—O1	1.241 (3)	C6—H6B	0.9600
C1—C2	1.408 (4)	C6—H6C	0.9600
			
C1—N1—C5	123.2 (2)	C2—C3—C4	122.3 (3)
C1—N1—H1	118.4	C2—C3—H3	118.8
C5—N1—H1	118.4	C4—C3—H3	118.8
C1—N2—H2A	120.0	C5—C4—C3	116.7 (2)
C1—N2—H2B	120.0	C5—C4—C6	121.4 (3)
H2A—N2—H2B	120.0	C3—C4—C6	121.8 (3)
O3—N3—O2	120.3 (3)	C4—C5—N1	121.2 (3)
O3—N3—O1	120.3 (3)	C4—C5—H5	119.4
O2—N3—O1	119.4 (2)	N1—C5—H5	119.4
N2—C1—N1	119.9 (3)	C4—C6—H6A	109.5
N2—C1—C2	123.1 (3)	C4—C6—H6B	109.5
N1—C1—C2	116.9 (2)	H6A—C6—H6B	109.5
C3—C2—C1	119.6 (3)	C4—C6—H6C	109.5
C3—C2—H2	120.2	H6A—C6—H6C	109.5
C1—C2—H2	120.2	H6B—C6—H6C	109.5
			
C5—N1—C1—N2	−179.3 (3)	C2—C3—C4—C5	−1.0 (4)
C5—N1—C1—C2	0.4 (4)	C2—C3—C4—C6	179.9 (3)
N2—C1—C2—C3	179.5 (3)	C3—C4—C5—N1	1.1 (4)
N1—C1—C2—C3	−0.2 (4)	C6—C4—C5—N1	−179.8 (3)
C1—C2—C3—C4	0.6 (4)	C1—N1—C5—C4	−0.8 (4)

### Hydrogen-bond geometry (Å, º)

**Table:**

*D*—H···*A*	*D*—H	H···*A*	*D*···*A*	*D*—H···*A*
N1—H1···O2^i^	0.86	1.95	2.808 (4)	177
N1—H1···O3^i^	0.86	2.53	3.122 (4)	127
N2—H2*A*···O1	0.86	2.18	2.992 (4)	157
N2—H2*B*···O1^ii^	0.86	2.12	2.948 (4)	160
C2—H2···O3^ii^	0.93	2.45	3.304 (4)	153

Symmetry codes: (i) *x*−1/2, −*y*+1/2, *z*−1/2; (ii) *x*+1/2, −*y*+1/2, *z*+1/2.
